# Knock-on effect of periodontitis to the pathogenesis of Alzheimer’s disease?

**DOI:** 10.1007/s00508-020-01638-5

**Published:** 2020-03-25

**Authors:** Friedrich Leblhuber, Julia Huemer, Kostja Steiner, Johanna M. Gostner, Dietmar Fuchs

**Affiliations:** 1 Department of Gerontology, Kepler University Clinic, Linz, Austria; 2 Freelance Certified Dental Hygienist, Linz, Austria; 3 Division of Medical Biochemistry, Biocenter, Innsbruck Medical University, Innsbruck, Austria; 4 Division of Biological Chemistry, Biocenter, Innsbruck Medical University, Innrain 80, 4th Floor, Room M04-313, 6020 Innsbruck, Austria

**Keywords:** Alzheimer’s dementia, Cognitive decline, Neopterin, Neuroinflammation, Pathogenic oral bacteria

## Abstract

**Background:**

Alzheimer’s disease has chronic inflammatory components, which can be enhanced by systemic immune activation resulting in inflammation or vice versa*.* There is growing evidence that chronic periodontitis drives systemic inflammation and finally Alzheimer’s disease. Thus, a link might exist between oral pathogens and Alzheimer’s disease. This may be of special significance as there is an age-related incidence of chronic periodontitis.

**Methods:**

In this study, 20 consecutive patients with probable Alzheimer’s disease were investigated. Diagnosis was established by cognitive tests, routine laboratory tests and cerebral magnetic resonance tomography. In 35% of these patients with cognitive impairment pathogenic periodontal bacteria were found.

**Results:**

The presence of* Porphyromonas gingivalis*, the key pathogen and one of the species involved in chronic periodontitis, was found to be associated with lower mini mental state examination scores (*p* < 0.05) and with a tendency to lower scores in the clock drawing test (*p* = 0.056). Furthermore, association between lower serum concentrations of the immune biomarker neopterin and the presence of *Treponema denticola *(*p* < 0.01) as well as of kynurenine were found in Alzheimer patients positive vs. negative for *Tannerella forsytia *(*p* < 0.05).

**Conclusions:**

Data indicate a possible association of specific periodontal pathogens with cognitive impairment, *Treponema denticola* and *Tannerella forsytia* may alter the host immune response in Alzheimer’s disease. Albeit still preliminary, findings of the study may point to a possible role of an altered salivary microbiome as a causal link between chronic periodontitis and cognitive impairment in Alzheimer’s disease.

## Introduction

In Alzheimer’s disease (AD), the most common form of sporadic dementia with a complex multifactorial etiology including periodontal bacteria [1], neuroinflammation is an early event which can be exacerbated by systemic inflammation [2]. Increased neopterin production and tryptophan breakdown were found to be sensitive biomarkers of immune activation, which are derived from stimulation of indoleamine 2,3-dioxygenase‑1 (IDO) and GTP(guanosine triphosphate)-cyclohydrolase 1 by interferon-gamma [3, 4]. Similar changes albeit to a lower degree can be observed in a large percentage of older individuals due to the age-related immune response even if healthy [5], but more often and to a greater extent in AD [6–8]. Especially the tryptophan metabolism could play a major role because the decrease of tryptophan concentrations and the parallel increase of the neurotoxic tryptophan catabolite quinolinic acid were observed to relate to impaired cognitive abilities of patients [2, 8].

A high percentage of older people suffer from periodontitis, the prevalence of which increases with age [9]. Interestingly, periodontal disease was found to be associated with higher brain amyloid load even in healthy older persons [10]. In recent studies, a close and causal link between chronic periodontitis, aggravating systemic inflammation and cognitive impairment was hypothesized [11, 12]. This hypothesis is underlined by recent preclinical data [13].

*Porphyromonas gingivalis* is described as the key pathogen which causes polymicrobial synergy and dysbiosis gaining greater virulence [14]. Different periodontopathogenic bacterial strains are described to correlate with inflammatory mediators and AD [15].

Dysbiosis of intestinal microbiota in old people has earlier been described to cause leaky gut, which results in silent systemic inflammation and via the microbiota-gut-brain axis in neuroinflammation, a relevant pathomechanism in the course of AD [2, 16–19]. Poor dental status and periodontal disease has earlier been linked to reduced cognitive function and AD [20]. In the Nun study, participants with the fewest teeth had the highest risk of prevalence and incidence of dementia [21, 22]. In an earlier postmortem study, pathogenic periodontal disease bacteria, *Treponema denticola, Tannerella forsythia, *and *Porphyromonas gingivalis*, were identified in brain tissue indicating a link between chronic periodontal disease and AD [23].

## Patients, material and methods

After written informed consent, the diagnosis of primary degenerative dementia (F 00.1, in accordance with the International Classification of Diseases-ICD-10) was established in 20 patients by cerebral magnetic resonance imaging (MRI) and routine laboratory tests as described earlier [19, 24]. Cognitive testing was performed by mini mental state examination (MMSE) and clock drawing test (CDT).

The following laboratory parameters were also analyzed: serum concentrations of neopterin and of tryptophan and kynurenine, calculating the kynurenine to tryptophan ratio (Kyn/Trp) as an index of tryptophan breakdown [4, 24]. Additionally, alveolar fluid was tested by RNA-based analysis (PerioPOC®, Genspeed Biotech, Henry Schein Dental, Vienna, Austria) for the presence of the following periodontal pathogens: *Treponema denticola, Tannerella forsythia, Porphyromonas gingivalis, Prevotella intermedia *and* Aggregatibacter actinomycetemcomitans *[25].

Data were analyzed by the Statistical Package for Social Science (version 22, IBM Statistics SPSS, Chicago, IL, USA). To take into account that not all collected data followed a normal distribution, non-parametric Friedman and Wilcoxon signed-rank test were applied. To test for associations between variables, Spearman rank correlation analysis was performed, and *p* values below 0.05 were considered to indicate statistical significance.

## Results

From 55 outpatients of the Department of Gerontology of the Neuromed Campus at the Kepler University Clinic Upper Austria with different neuropsychiatric symptomatology, 20 consecutive patients (aged 78.1 ± 2.2 years, 9 females) with symptoms of cognitive impairment were recruited. None of them were smokers.

The procedure was well-tolerated by all patients. In MRI scans all patients showed global cerebral atrophy without circumscribed vascular lesions. The mean ± SD MMSE in the patients was: 20.5 ± 7.0 and the CDT scores were 5.8 ± 3.1.

In seven of the patients investigated with clinical signs of periodontitis stage III and IV [26], pathological periodontitis strains were found: *T. forsythia *in one*, T. denticola *and* T. forsythia *together in one,* T. denticola, T. forsythia *and* P. gingivalis* together in five patients; these also showed the lowest MMSE and CDT scores, see Table 1. *P. intermedia *and* A. actinomycetemcomitans *could be detected in none of the cases studied.

**Table 1:** Clinical characteristics, psychometric and immunological data in 20 AD patients tested for periodontopathological bacteria

Patient	Sex	Age	Trp	Kyn	K/T	Neo	Trep‑d	Tann‑f	Por‑g	MMSE	CDT
ID		(years)	µM	µM	µM/mM nM						
KR	Male	86	56.4	1.84	32.7	12.5	0	0	0	23	7
KR	Female	63	71.0	2.08	29.3	9.6	0	0	0	29	7
KP	Male	86	51.3	1.53	29.9	8.5	0	0	0	25	9
AG	Male	79	58.4	1.11	19.1	4.5	0	0	0	27	9
GR	Male	76	56.0	1.91	34.2	7.5	0	0	0	25	5
IL	Male	74	43.4	1.31	30.3	6.1	1	1	0	24	7
FB	Male	79	57.2	2.53	44.2	7.8	1	1	1	3	3
AZ	Female	92	58.6	1.76	30.0	5.9	0	0	0	14	3
JS	Female	78	51.8	2.03	39.1	9.3	0	0	0	17	7
WT	Male	72	57.5	1.81	31.4	8.2	0	0	0	29	9
SF	Male	87	70.4	3.58	50.9	14.0	0	0	0	21	7
RN	Female	86	53.0	2.09	39.4	8.5	0	0	0	21	7
JH	Male	73	73.0	2.86	39.2	13.6	0	0	0	27	9
WP	Male	72	48.3	1.29	26.6	4.9	1	1	1	18	3
KR	Male	76	62.8	1.76	28.0	7.4	1	1	1	8	0
MM	Female	73	69.7	1.77	25.4	8.9	0	1	0	25	7
MB	Female	56	54.7	1.18	21.6	4.5	1	1	1	24	9
LH	Female	97	66.7	2.14	32.1	8.3	0	0	0	14	0
HP	Female	69	36.6	1.67	45.7	13.3	0	1	1	14	0
TH	Female	87	53.1	3.38	63.6	11.1	0	0	0	21	7
Mean	–	78.1	57.5	1.98	34.6	8.7	–	–	–	20.5	5.8

*Trp* Tryptophan, *Kyn* Kynurenine, *K/T* Kynurenine/tryptophan, *Neo* Neopterin, *Trep‑d* Treponema denticola, *Tann‑f* Tannerella forsythia, *Por‑g* Porphyromonas gingivalis, *MMSE* mini mental state examination, *CDT* clock drawing test

**Fig. 1:**
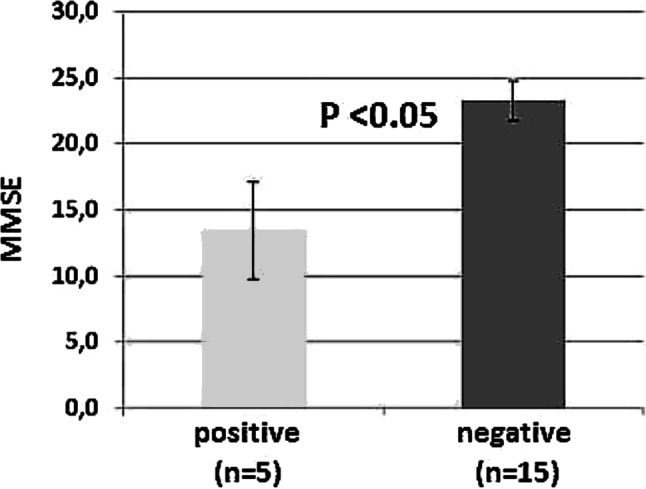
Patients with AD positive for *Porphyromonas gingivalis* present with statistically lower scores in the mini mental state examination test (MMSE; left)

As a main result in this investigative study, a significant association was observed between the salivary presence of *P. gingivalis* and lower MMSE (positive: 13.4 ± 3.68; vs. negative: 23.3 ± 1.50; U = 2.239, *p* < 0.05; Fig. 1). There was also a tendency to lower scores in the CDT (positive: 3.00 ± 1.64; vs. negative: 7.1 ± 0.73; U = 1.989, *p* = 0.056; Table 1) when this particular pathogen was present.

**Fig. 2:**
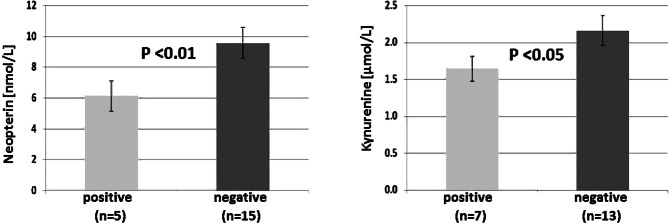
Lower mean values ± SEM of serum neopterin (*left*) and kynurenine (*right*) concentrations in AD patients positive for *Treponema denticola* (*left*) and *Tannerella forsythia* (*right*) respectively

Furthermore, the presence of* T. denticola *was associated with lower serum neopterin concentrations (positive: 6.14 ± 0.65 vs. negative: 9.58 ± 0.73 nmol/L; U = 2.533, *p* < 0.01); the presence of *T. forsythia *resulted in lower serum kynurenine concentrations compared to a negative saliva test result (positive 1.64 ± 0.17 vs. negative: 2.16 ± 0.20; U = 1.980, *p* < 0.05; Fig. 2; Table 1).

In the whole data set, a significant positive correlation existed between serum concentrations of neopterin and Kyn/Trp (rs = 0.674, *p* < 0.001) confirming earlier results [2, 27].

## Discussion

Results of the present series confirm that chronic low-grade immune activation and inflammation alter monoamine metabolism that may be involved in the development of neuropsychiatric symptoms characteristic for age and dementia [2, 5, 27, 28]. The present exploratory pilot study described the presence of the periodontal pathogenic bacteria *T. denticola, T. forsythia, *and *P. gingivalis* in saliva of a subgroup of demented patients causing chronic oral inflammation as mentioned earlier [19, 29]. *P. gingivalis*, the most virulent pathogen of the bacteria associated with periodontitis [14] with remote body inflammatory pathologies, was found to be associated with lower MMSE (*p* < 0.05; Fig. 1) and also albeit only weakly with lower CDT scores (*p* < 0.06) in the patients indicating a possible link between this periodontal pathogenic strain and neuroinflammation and the dementia process.

In an animal study published recently [13], experimental chronic periodontitis was induced by repeated oral application of *P. gingivalis*. Gingipain, a protease secreted by this bacterium, could be detected immunohistochemically in the hippocampi of experimental mice confirming its translocation to the brain. Also, signs of neuroinflammation, neurodegeneration and the formation of extracellular Aß_42_ (myloid -peptide 42) consistent with neuropathological findings in AD could be shown after repeated oral application of *P. gingivalis* in young adult wild type mice [13]. In AD, *P. gingivalis* possibly affects the blood-brain barrier permeability and influences local IFN‑γ response by preventing entry of immune cells into the brain. The scarcity of adaptive immune cells in AD neuropathology implies *P. gingivalis* infection of the brain likely causing impaired clearance of insoluble amyloid and inducing immunosuppression [14, 30]. An inverse association was seen between the presence of salivary *T. denticola* and concentrations of neopterin (*p* < 0.01) as well as between salivary *T. forsythia* and kynurenine (*p* < 0.05). These results probably indicate that these pathogens may counteract adaptive (Th1 type [T helper cells type 1]) immunity by triggering regulatory T‑cells which finally may suppress and downregulate the inflammation process as recently hypothesized [2, 20]. Likewise, *P. gingivalis* was reported to suppress adaptive immunity in AD patients [30]. Probably an imbalance of immune functions and an imbalance of adaptive immunosuppression could be caused by these pathogenic bacterial species; however, associations between the presence or absence of periodontal pathogens and abnormal concentrations of immune system biomarkers neopterin and/or kynurenine were only seen for 3 pathogens, namely *T. denticola, T. forsythia *and* P. gingivalis, *but not for *P. intermedia *and* A. actinomycetemcomitans. *This result, however, can only be regarded as preliminary because of the small number of patients investigated thus far.

Interestingly, six of the patients positive for periodontal pathogens were ApoE4 (apolipoprotein E4) allele carriers, two of them homozygous; in an earlier study, individuals with both a low number of teeth and the ApoE4 allele performed worse in cognitive tests an cd showed more rapid cognitive decline with time [31]. The data set in this study was too small for detailed statistical analysis, and future studies will be necessary to address this issue properly.

To conclude, in the absence of longitudinal data the preliminary findings can only provide correlational evidence that periodontal bacterial infections may be additive in the pathogenesis of cognitive impairment and AD. Any correlation—even if significant—cannot be interpreted as cause-effect relationship in any direction. It only indicates an association between the events. Future longitudinal studies including periodontal disease in larger numbers of dementia patients as well as age-related non-dementia individuals [10] with respect to the ApoE status are necessary to elucidate the specific role of the oral microbiome in neuroinflammation and neurodegeneration and its potential to prevent or delay the onset of AD. The presence of specific pathogens relating to immunobiochemical changes in patients could probably reflect the fact that these patients are no longer able to perform sufficient dental hygiene. Additional longitudinal studies also should investigate—beyond sufficient dental hygiene [14]—the effects of the consumption of a certain bacterial strain to improve or prevent periodontal disease in the aging population [32]. Small molecule inhibitors to reduce the bacterial load of certain bacteria like *P. gingivalis* will be developed in the near future [33]. Thus, encompassing investigation of the symbiotic intestinal, oral as well as nasal microbiome might give additional information to define new pathways for evolutionary AD treatment [34].

This study is certainly limited by the relatively small number of patients. Thus, results can only be regarded as preliminary; however, they provide some new aspect that the salivary microbiome could play a relevant role in the pathophysiology of AD. It might also draw attention to a potential bottom-up contribution in AD which is usually regarded to be top-down only. With this respect it appears attractive that saliva can be easily assessed with no burden to the patients but enables monitoring of a potentially important aspect in the pathophysiology of AD.
