# Correction to: Knock-on effect of periodontitis to the pathogenesis of Alzheimer’s disease?

**DOI:** 10.1007/s00508-020-01647-4

**Published:** 2020-04-06

**Authors:** Friedrich Leblhuber, Julia Huemer, Kostja Steiner, Johanna M. Gostner, Dietmar Fuchs

**Affiliations:** 1 Department of Gerontology, Kepler University Clinic, Linz, Austria; 2 Freelance Certified Dental Hygienist, Linz, Austria; 3 Division of Medical Biochemistry, Biocenter, Innsbruck Medical University, Innsbruck, Austria; 4 Division of Biological Chemistry, Biocenter, Innsbruck Medical University, Innrain 80, 4th Floor, Room M04-313, 6020 Innsbruck, Austria

**Correction to:**

**Wien Klin Wochenschr 2020**

10.1007/s00508-020-01638-5

The original version of this article unfortunately contained a mistake. There was an error in Fig. 2 as well as an error in two of the values. It should read *p* < 0.05 instead of *p* < 0.03 in the sections Summary (line 8) and Results (line 26). The original article has been corrected. We apologize for the mistake.

The correct Fig. 2 is given below.

**Fig. 2:**
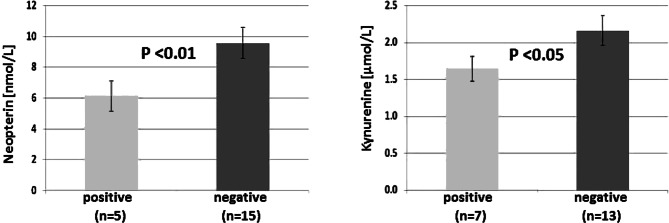
Lower mean values ± SEM of serum neopterin (*left*) and kynurenine (*right*) concentrations in AD patients positive for *Treponema denticola* (*left*) and *Tannerella forsythia* (*right*) respectively

